# Gaze when walking to grasp an object in the presence of obstacles

**DOI:** 10.1167/jov.25.11.12

**Published:** 2025-09-18

**Authors:** Dimitris Voudouris, Eli Brenner

**Affiliations:** 1 Experimental Psychology, Justus Liebig University Giessen, Germany; 2 Department of Movement Sciences, Vrije Universiteit Amsterdam, The Netherlands

**Keywords:** grasping, gait, navigation, obstacles, gaze

## Abstract

People generally look at positions that are important for their current actions, such as objects they intend to grasp. What if there are obstacles on their path to such objects? We asked participants to walk into a room and pour the contents of a cup placed on a table into another cup elsewhere on the table. There were two small obstacles on the floor between the door and the table. There was a third obstacle on the table near the target cup. Participants often looked at the items on the table from the beginning, but, as they approached and entered the room, they often looked at the floor near the obstacles, although there was nothing particularly informative to see there. Thus they primarily relied on peripheral vision and memory of where they had seen obstacles to avoid knocking over the obstacles. As they approached the table, they mainly looked at the object that they intended to grasp and the obstacle near it. We conclude that people mainly look at positions at which they plan to physically interact with the environment, rather than at items that constrain such interactions.

## Introduction

When performing their daily actions, humans typically direct their gaze to positions of interest for the ongoing or upcoming movement. This is well documented for various tasks, from isolated pointing (Neggers & Bekkering, 2000) and grasping (Brouwer, Franz, & Gegenfurtner, 2009) movements, to the sequential actions that are required to prepare breakfast (Land, Mennie, & Rusted, 1999), take part in sports (Mann, Spratford, & Abernethy, 2013), steer a car (Tuhkanen, Pekkanen, Wilkie, & Lappi, 2021), and navigate staircases (Ghiani, Van Hout, Driessen, & Brenner, 2023) or rough terrain (Matthis, Yates, & Hayhoe, 2018). Gaze shifts bring relevant positions of the environment onto the fovea, which is the part of the retina with the highest spatial resolution. People typically direct their gaze at the target of their movement before initiating a movement toward that target (Johansson, Westling, Backstrom, & Flanagan, 2001; Hollands & Marple-Horvat, 2001), and keep fixating the target until reaching it (Brenner & Smeets, 2007; Cámara, de la Malla, López-Moliner, & Brenner, 2018), which allows them to use the best possible visual information to both plan and guide the movement to the target. Relying on visual input with the highest possible spatial resolution is presumably important in demanding tasks that require high spatial and temporal precision, such as grasping an object (Voudouris, Smeets, & Brenner, 2016) or intercepting moving objects (Brenner & Smeets, 2011).

In many tasks, it is evident where the relevant information is to be found, but there are also tasks in which there are multiple important positions, so one needs to supplement information from where one is looking with information from other sources, such as peripheral vision or memory (Franchak & Adolph, 2010). An example is when simultaneously reaching with both hands to different targets, in which case people look at the target that requires a higher degree of visual guidance, such as the smaller target or the target that one is reaching for with the non-dominant hand (Riek, Tresilian, Mon-Williams, Coppard, & Carson, 2003). When reaching to grasp a single object in the presence of obstacles, humans primarily fixate the target object rather than the obstacle (Grant, 2015; Marotta & Graham, 2016; Voudouris et al., 2016), suggesting that it is important to have precise information about the target object, while obstacles can be cleared without looking directly at them, possibly because peripheral vision is precise enough to monitor obstacles’ positions relative to the moving arm. When tracking or intercepting targets with a cursor, people are known to look at the target rather than the cursor (Cámara et al., 2018; Danion & Flanagan, 2018), relying on information from peripheral vision and kinaesthesia to guide the cursor to the target. That humans can perform tasks without looking at all relevant positions is evident from the fact that they can successfully climb staircases while looking at their phones rather than at the steps, though they do slow down their stair climbing to do so (Ioannidou, Hermens, & Hodgson, 2017). This suggests that humans use peripheral vision to guide their steps, or that they simply replicate previous, successful stride sizes. Likewise, when navigating flat terrains, humans often look straight ahead, away from potential footholds, but on rough, more complex terrains, gaze is primarily directed to upcoming footholds (Matthis et al., 2018; ‘t Hart, Schmidt, Klein-Harmeyer, & Einhaeuser, 2013). People also look longer at their next footholds when the positions of such footholds are less clearly defined (Dominguez-Zamora, Gunn, S. M., & Marigold, 2010). When having to step over obstacles of different heights that are obstructing the walking path, people spend more time looking at the obstacles when the obstacles are higher (Patla & Vikers, 1997), showing that as the stepping accuracy demands increase, people rely more on central vision to guide their leg. All in all, there appears to be a close coupling between gaze allocation and body movements during continuous actions, whereby gaze is presumably directed in the manner that best ensures that all aspects of the action are performed successfully.

We here examine gaze patterns when people walk into a room to grasp an object. In particular, we examine how gaze is influenced by obstacles on their walking path. Previous work has shown that humans sequentially look at objects that they need to reach and at obstacles that they need to avoid when walking through a virtual scene with many objects and obstacles along their path (Tong, Zohar, & Hayhoe, 2017). Thus we might expect participants to fixate each obstacle as they approach it, only fixating the object that they need to grasp after having passed the floor obstacles, especially if the object that they need to grasp is always at the same position in the room. We placed an obstacle near the target object to make it more difficult to reach out and grasp the target object, making it more likely that people will look at it, and at the nearby obstacle, for extended periods of time. We also had people move the target object in different directions after grasping it, so they might also want to look where they should move the target object as they approach it. If people mainly look at such items related to grasping the target as they walk into the room, they have to rely on peripheral vision and possibly on memory of the positions of any obstacles on the floor to guide their steps away from such obstacles along their walking path. Such memory of the obstacles’ positions would have to be based on visual information obtained from earlier glimpses at the obstacles when entering the room, because the obstacles’ positions were not the same every time the person walked into the room. The variability in obstacle positions also means that participants could not simply repeat their actions. Peripheral vision probably has a high enough resolution for avoiding the conspicuous obstacles (Cámara et al., 2018), even when it is not complemented by estimates of obstacles’ positions based on earlier views of the scene. We therefore wanted to know whether people would primarily fixate the target object, and possibly other items related to the grasping task, relying on peripheral vision to guide their foot placement, or whether they would often fixate obstacles along their walking path or the places at which they intend to place their feet when passing such obstacles.

Participants walked into a room to grasp a cup of water placed at a fixed position on a table. There were two obstacles (other cups) on the floor along the walking path, and a third obstacle (yet another cup) on the table near the target object. Upon grasping the cup of water, participants had to empty its contents into a final cup that could be at either end of the table. If gaze is allocated sequentially, participants should look at positions of interest as these positions become relevant. They might start by looking at all five cups as soon as they can to determine the most efficient walking path and grasping posture considering all constraints imposed by the obstacles. They should then first look at the first and then at the second obstacle on the floor to make sure to avoid knocking them over. After crossing these obstacles, they should look at the obstacle next to the cup of water and the cup of water itself to plan the most suitable grasping posture. Finally, they should look at the cup into which they are going to pour the water. But alternatively, they might quickly direct their gaze at the cup of water and nearby obstacle, where the required precision is highest, and rely completely on peripheral vision to avoid knocking over the obstacles on the floor. A sequential pattern has been found in continuous tasks such as walking (Tong et al., 2017), while maintaining gaze where most precision is required is the typical behavior in discrete tasks such as grasping when standing still (Voudouris, Smeets, Fiehler, & Brenner, 2018). Most studies examining gaze allocation during grasping had participants sit or stand at a position from which they only had to move their arm to reach and grasp the object (Brouwer et al., 2009; Grant, 2015; Voudouris et al., 2016; Voudouris et al., 2018). Here, we examine how gaze is allocated when people have to take a few steps to grasp an object. Will they still mainly look at the object they intend to grasp, or will they first look at the obstacles on the floor?

## Methods

### Participants

Ten participants volunteered to take part in the experiment (six women; age range 21–33, median age = 26; height range 160–196 cm, median height = 170 cm; eye height range, 149–182 cm, median eye height = 158 cm). They had normal or corrected-to-normal vision, were right-handed by self-report, and were free from any known neurological or musculoskeletal issues that would make it difficult for them to walk or to grasp and manipulate a cup. Participants signed informed consent forms prior to taking part in the experiment. The study was approved by the local ethics committee of Justus Liebig University Giessen and was conducted in accordance with the Declaration of Helsinki (2013; except for §35, pre-registration). Participants were compensated with €8/hour or with course credits for their efforts.

### Setup and data collection

**Figure 1.**
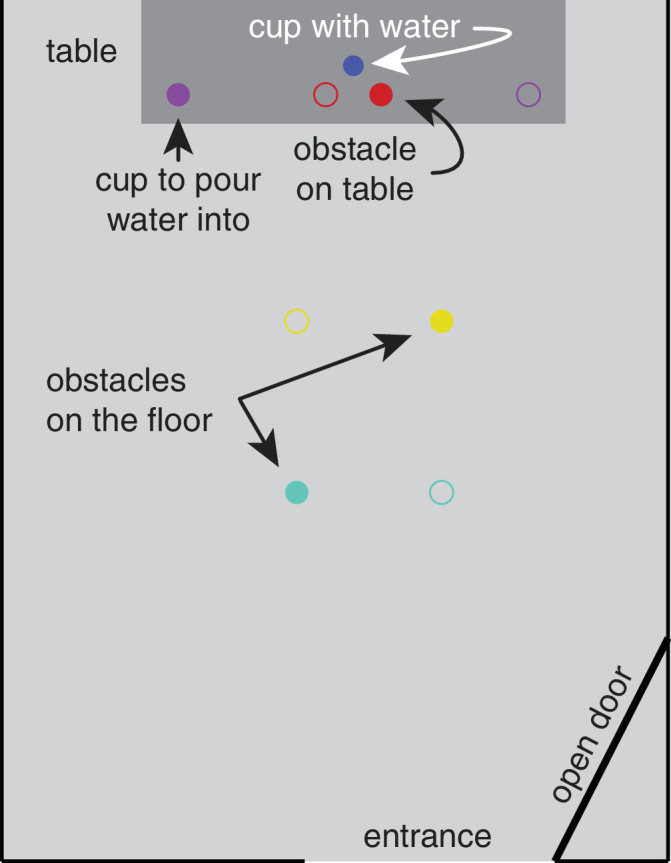
Schematic representation of a top view of the room, with the (open) door through which the participant entered the room at the bottom and the table at the top. The room extended beyond the top, but otherwise the representation is drawn to scale. The distance between the entrance and the near edge of the table was 280 cm. The task was to pick up the blue cup of water and pour its contents into the purple cup, while not knocking over the two obstacles on the floor along the path (turquoise and *yellow* cups), or the obstacle (*red* cup) near the cup of water. Open circles represent the alternative position of the obstacles and destination cups. The colors represent the actual colors of the cups we used in the experiment.

Participants were asked to walk into a room (296 × 480 cm), pick up a cup of water placed on a table (161 × 80 cm; surface at 71 cm from the floor), and pour the water into another cup placed at one of two corners of the table. Participants started each trial standing in a hallway about 3 m from the door to the room. They were instructed to perform the task without knocking over three more cups: the obstacles. Two of these obstacles were on the floor along their walking path and the last one was near the target cup on the table. Thus, in total, there were five cups: the target cup, the destination cup, and the three obstacles (Figure 1). The target cup (the cup with the water) was placed centrally on the table, 22 cm from the edge that was closest to the participant. The destination cup was placed 66.5 cm to the left or right of the target cup, 11 cm from the edge of the table that was closest to the participant. Participants had to grasp and manipulate the cup using their right thumb and index finger. The table was positioned so that its near edge was 280 cm from the entrance to the room. The two floor obstacles were placed inside the room and were positioned at 140 and 205 cm from the door. Each floor obstacle could be placed at one of two lateral positions that were 55 cm apart. These positions were chosen to be close enough to the straight path to the table to be relevant for approaching the table, but far enough apart for participants to be able to place their foot between them and for us to be able to tell which of the two obstacles participants were looking at. The table obstacle was placed 11 cm to the side of and 11 cm in front of the target cup (aligned with the destination cup). All objects were plastic cups. They were 15.5 cm tall, with a 9 cm mouth diameter and a 6 cm base diameter, except the target cup that was 9 cm tall, with an 8 cm mouth diameter and a 5.3 cm base diameter. Each of the five cups had a different color to make it easier to recognize the cups in the scene, which was important for extracting the cups’ positions from the data from the eye tracker's scene camera to relate them to gaze, and for estimating the participant's position (see Data Analysis). A schematic depiction of the setup and of the possible item configurations is shown in Figure 1.

Participants wore a portable eye tracker (Pupil Invisible glasses; Pupil Labs, GmbH, Berlin, Germany) that consists of a spectacle frame holding cameras that record images of the eyes and of the scene. The eye tracker is connected with a cable to a smartphone, on which the data is stored. The scene camera is attached to the left side of the spectacle frame and has a field of view of 82° × 82°. Scene images were recorded at an average rate of 26 Hz (range 25–27 across participants). The eye tracker records eye movements at about 200 Hz and then uses deep learning to directly estimate gaze from the recorded eye images. Having been trained on a large and diverse dataset, it provides a good estimate of gaze position under variable conditions without requiring any calibration. A description of this process and of the glasses’ performance can be found at arxiv:2009.00508. Additional tests of the performance of our particular tracker can be found in Ghiani, Amelink, Brenner, Hooge, and Hessels (2024). We resampled the eye tracker data at the precise times for which we had scene images using local polynomial interpolation (Savitzky-Golay filter with a window of 100 ms).

For each participant, we first started the recording of the eye tracker and then placed the recording phone in his or her pocket. Care was taken that the connecting cable would not hinder the participants’ walking and grasping movements. The participant started each trial standing in a hallway, about 3 m from the door, facing away from the room. The starting position was visually aligned with a mark on the hallway, so there is some variability across trials and participants. Meanwhile, one of the experimenters placed all five cups at the appropriate positions for the upcoming trial, and the other experimenter stood next to the participant holding a black card in front of the eye tracker's scene camera. Occluding the scene camera made it easy to later segment the videos into separate trials (see Data Analysis). Once the configuration was ready, the experimenter removed the black card, which was the go-cue for the participant to start the trial. Participants then rotated their body and walked into the room to perform the task. No specific instructions were given other than that participants should grasp and manipulate the target cup using the thumb and index finger of their right hand, and that they should be careful not to knock over any obstacle and not to spill the water. Once participants had performed the task, they walked back out of the room to the experimenter with the black card and waited for the next trial to begin.

Each combination of the 16 possible configurations (two near floor obstacle positions, two far floor obstacle positions, two table obstacle positions, two destination cup positions) was presented five times for a total of 80 trials. We presented these configurations in random order to discourage participants from repeating the same set of walking and grasping actions over consecutive trials. Walking into the room and performing the grasping and manipulation task took about 12 seconds. Including walking back out of the room and waiting for the experimenter to set up the next configuration (and pour the water back into the target cup), a trial took about 22 seconds, so the whole experiment lasted about 35 minutes.

### Data analysis

**Figure 2.**
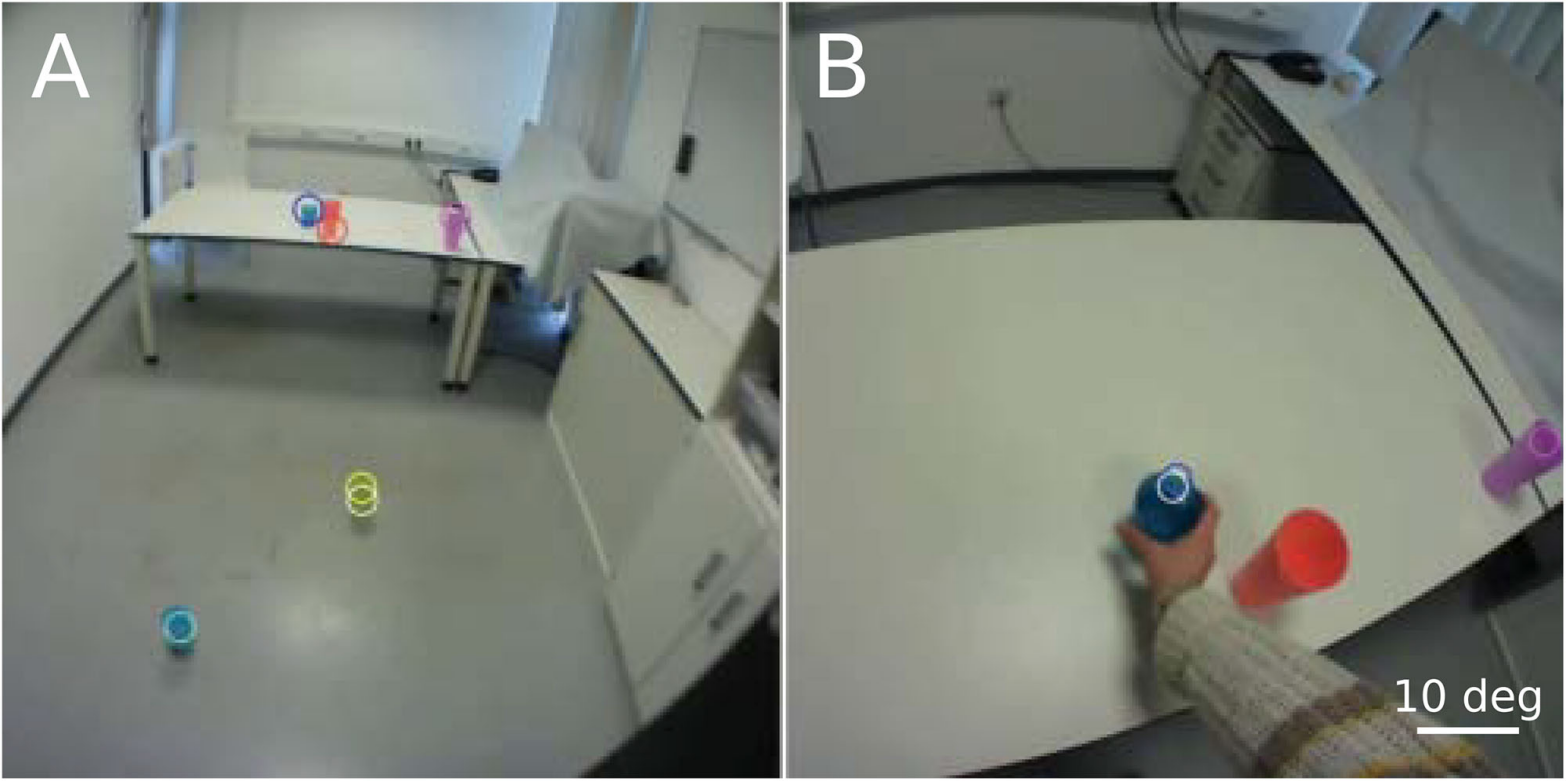
Scene camera view when the participant (**A**) enters the room and (**B**) picks up the target cup. The items in the scene are shown together with their estimated positions (circles of corresponding color) based on our analysis. The white circles indicate where the participant is looking on these frames according to the eye tracker: at the yellow cup in **A** and the blue one in **B**.

The eye tracker provides information about gaze in the scene, but does not identify the items in the scene. Using differently colored cups allowed us to identify the relevant items by their color. Relying only on color, light falling on the floor was sometimes mistaken for the yellow cup. To prevent this from happening, the software was designed to only look for cups at plausible positions relative to each other and to their positions on the previous image frame. Two snapshots from a single trial together with the identified cup positions are shown in Figure 2. We also used the identified scene positions of all visible cups together with the participant's eye height to estimate where the participant (scene camera) was at that time. This could be done as long as all the cups on the table and at least one of the cups on the floor was within the scene. We determined the participant's most likely position from which the configuration in the room would give that image (for that participant's eye height). It is obviously not very precise, because small offsets of the identified cup positions from the centers of the cups in the scene, small deviations in where the cups were actually placed, and vertical displacements of the head during walking can all introduce small errors. To increase precision, we smoothed the estimates of the participant's distance from the table and of their lateral position across time (Savitzky-Golay filter with a window of 40 frames). The resulting estimates usually gave a reasonable idea of where the participant was, although occasionally this method resulted in strange paths (see Results), possibly because a cup was placed at the wrong position or some other item in the room was mistaken for a cup in some frames.

Automatic recognition of the configuration of the cups revealed that a cup was placed at a completely wrong position twice (in one case the red cup was placed at an irrelevant position on the table; in the other a cup must have been placed at the wrong side because rather than five trials for two of the configurations there were six for one and four for the other). These trials were included in the analysis. Because we determined which configuration was present on each trial from the images, the latter error in placing the cup is no problem. The former will give rise to a somewhat strange apparent walking path and therefore also a modest additional error in the gaze position on the floor (because the participant's position is considered when converting the angular distances between gaze and the two cups in the image to positions on the floor).

As a first step in our gaze analysis, we estimated the precision of our gaze estimates. To do so, we assumed that participants were looking at the water entering the purple cup while they were pouring the water, because at that time it is clear that the pouring action requires precision, and participants never spilt the water. We looked for periods of at least 10 frames (about 333 ms) from when the participant reached the table during which the blue (target) and purple (destination) cups were less than 5° apart, because this was only the case when participants were pouring the water. For each such period, we determined the average and standard deviation of the horizontal and vertical distances between gaze and the estimated center of the purple cup. We then determined the median value of these averages and standard deviations for each of the 10 participants. Finally, we averaged these median averages and standard deviations across participants. This resulted in average horizontal and vertical offsets of −0.5° and 3.9°, and average horizontal and vertical standard deviations of 1.8° and 4.0°. These standard deviations combine variability because of eye tracking with variability in our estimates of the position of the cup, which is the relevant measure of precision for our study. The horizontal offset is probably an artifact of the scene camera of the Pupil Invisible eye tracker being placed at the side of the glasses, which gives rise to a systematic parallax error when objects are nearby. The vertical offset is largely due to participants fixating the water entering the purple cup rather than the center of the purple cup. Consequently, they look at the top of the cup, or even above it. They probably also sometimes shift their gaze along the stream of water, and higher in the purple cup as it fills, which increases the variability in vertical position with respect to the purple cup.

Assuming that people normally look at items of interest, we consider gaze to be directed at a cup whenever the measurements do not suggest that it is unlikely that gaze is directed towards that cup. We consider it to be unlikely if gaze is more than about two standard deviations from the center of the cup, because the cups themselves extended less than 4° of visual angle (except when they were very nearby; Figure 2) and about 95% of the data should fall within 2 *SD* of where the participant is actually looking. The 2 *SD* corresponds with 3.6° in the horizontal direction. In the vertical direction the standard deviation is larger, but we know that the vertical standard deviation is an overestimate because of the way we determined it, as explained above, so we use an overall distance of 4° to decide whether gaze was directed toward each cup.

We next determined where participants were looking. We distinguished between 11 options. The first five options are that participants were looking within 4° of the center of one of the five cups (two floor obstacles, the table obstacle, the target cup, and the destination cup). Because the red and blue cups were close to each other, gaze was often within 4° of the center of both cups, so we categorize these instances as gaze toward “cups on table.” If none of the table cups is within 4° of the estimated gaze point, but gaze is higher than 5° below the blue cup and closer to the blue cup than the purple cup (or within 20° from the blue cup while the purple cup is not visible), we categorize gaze as being directed at the “table.” If none of the floor obstacles is within 4° from the estimated gaze point but gaze is lower than 5° below the blue cup, we categorize gaze as looking at the “floor.” In any other case, such as looking to the side or well above the table, gaze is characterized as looking “elsewhere.” Apart from these categories there are also moments at which participants blinked (“blink”), and moments at which we do not have reliable data (“none”). We plotted which of these options was valid at each estimated distance from the table for each trial of each participant, as well as on average. We considered distances from about 1.5 m from the door (so still well outside the room) until 50 cm from the blue cup (so more or less at the table). The reason for not analyzing gaze even earlier is that participants often started walking toward the door while looking at various, irrelevant places, such as the wall in front of them, a hallway to their left, or another room to their right. In such cases, not enough cups were available in the scene camera so we could not estimate the participant's position.

Combining our estimates of where the participants were with their gaze with respect to the positions of the obstacles on the floor at that moment, we could estimate where on the floor the participant was looking at each moment (when looking at the floor or the cups on the floor). We plot these positions, as well as the paths that participants took, separately for each of the four possible configurations of floor obstacles. To examine possible effects of the items’ configurations on the duration of gaze on the floor, we submitted individual participants’ fractions of time spent looking at the floor when between 1 m before entering the room and crossing the first obstacle to a 2 (near floor obstacle; turquoise cup) × 2 (far floor obstacle; yellow cup) × 2 (target obstacle; red cup) × 2 (destination cup; purple cup) repeated-measures analysis of variance.

## Results

**Figure 3.**
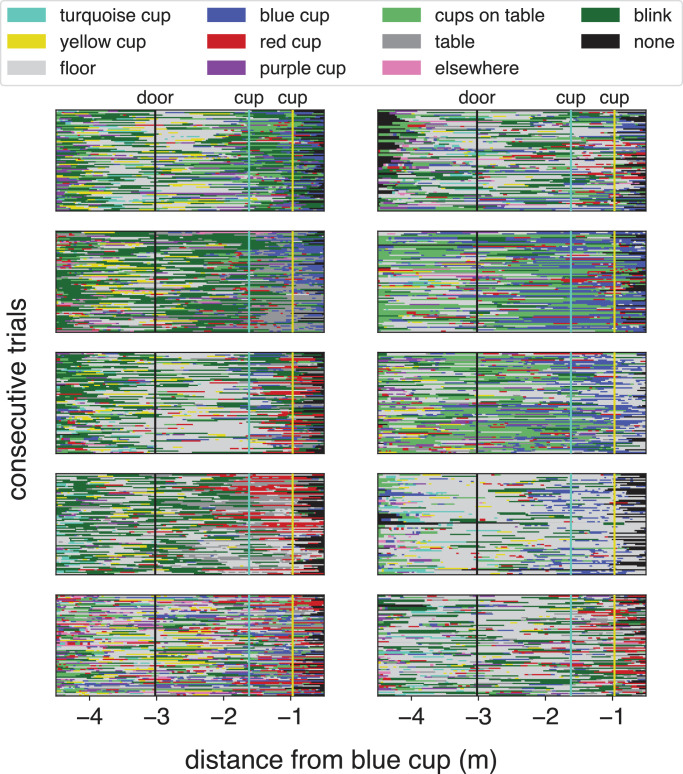
Items that participants were looking at on their way to the *blue* cup. Each panel represents one participant. Each row represents one trial, starting with the first trial at the bottom and ending with the last trial at the top.

Our participants never knocked over any cup; neither on the floor nor on the table. Neither did they ever fail to pour the water into the destination cup without spilling any of the water. There were systematic differences between where the participants looked, but they all often looked at the floor as they entered the room (Figure 3). Not surprisingly, they often looked at the table and the cups on the table as they got closer to the table. Participants also blinked. They differed in how frequently they blinked. At far and close distances there is also a category “none.” At far distances this is because participants oriented their head, and therefore the scene camera, in a manner that did not allow us to judge their distance from the blue cup, because too few cups were visible in the scene camera. In most cases they were probably looking elsewhere, such as at the wall near the door. Occasionally they turned so soon after removing the black card away from the scene camera that the camera was still adjusting to the transition between the dark image caused by the black card and the bright image of the room, in which case the cups also could not be detected reliably for one or two frames. At near distances, as the participant came close to the table, we often could no longer estimate their position reliably because too few cups were visible in the images. With only the cups on the table visible, the fact that these cups were more or less aligned made it impossible to reliably determine whether a change in their separation in the image was due to a change in viewing distance or a change in viewing angle (as a result of a lateral displacement). This is not a problem for any of the analyses, because when the participant is so close to the table they can only look at the cups on the table or at the table itself because they are standing close to the table so there is very little else to see. However, it means that the judged lateral position of the participant when very close to the table should not be taken too seriously.

**Figure 4.**
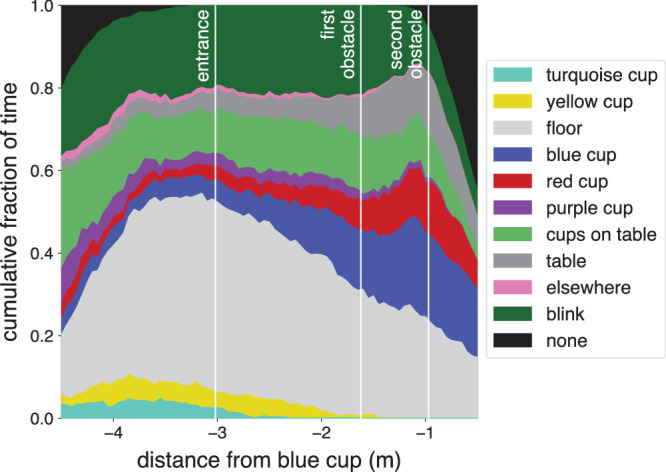
The fraction of time that participants were looking at different items in the scene. Averaged across all trials of all 10 participants.

Where participants were looking, on average, is clearer in Figure 4 that shows the overall distribution of where participants were looking as a function of their distance from the blue cup. The options are ordered systematically, so that the top of the light gray section indicates the fraction of time spent looking at the floor and cups on the floor, and the part between the top of the light gray area and the top of the dark gray area indicates the fraction of time spent looking at the table and cups on the table. At the beginning of the trial, before entering the room, participants often directed their gaze toward the table and cups on the table. As they entered the room they directed their gaze more frequently to the floor, and as they walked by the obstacles they looked at the table and cups on the table again. They blinked less as they approached the table, possibly in order to ensure that they have the best possible visual information when reaching out for the blue cup. One issue that we would like to point out is that while participants mainly looked at the cups when looking at the table (dark grey area is *smaller* than the sum of the blue, red, purple and light green areas), they mainly looked at the floor rather than the cups on the floor when looking at the floor (light grey area is *larger* than the sum of the yellow and turquoise areas, even well before passing the yellow and turquoise cups).

Thus gaze was directed towards regions that were relevant for planning how to pick up the target cup well in advance, rather than only being directed toward those regions after having made sure not to knock over the floor obstacles while walking towards the table. Moreover, participants did often look at the floor as they entered the room, but seldom directly at the obstacles on the floor. To examine the latter in more detail we look at the paths that participants take and where on the floor they generally look.

**Figure 5.**
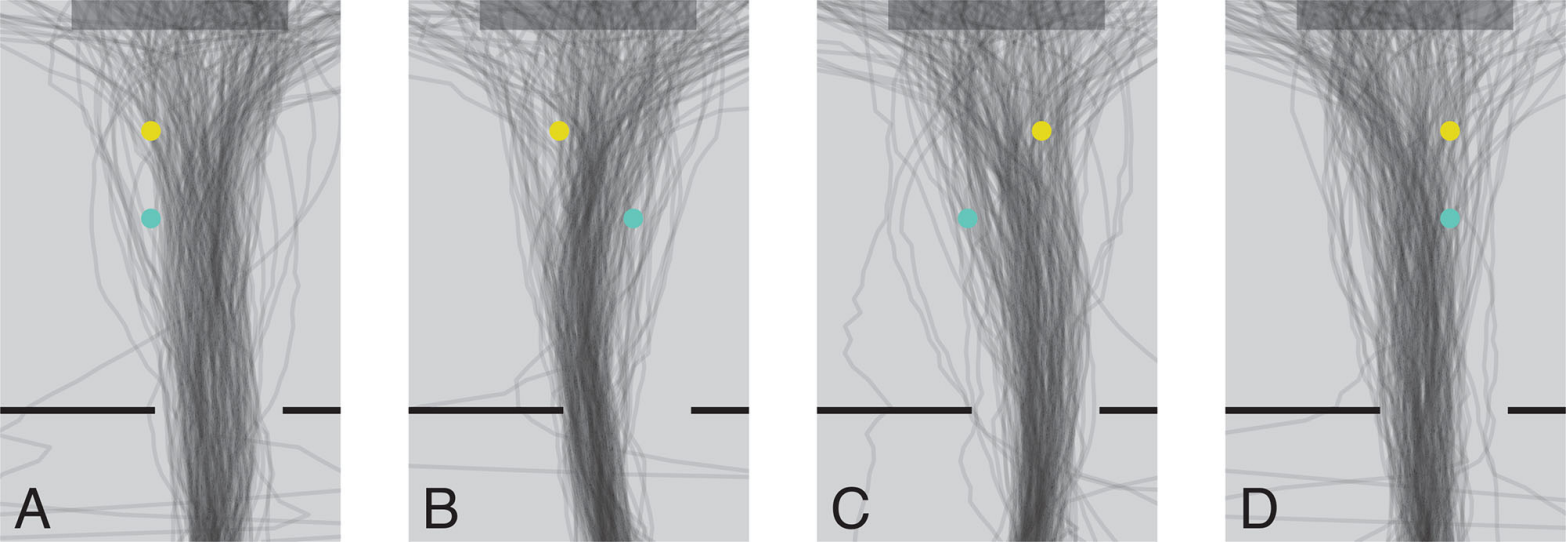
Estimated walking paths (lines) for each of the four possible floor obstacle configurations. Plots show a top view of the room, from the door (bottom) to the table (top). The *yellow* and turquoise disks are the two floor obstacles, so each of the panels **A**–**D** represents a single floor obstacle configuration. Each line represents a single trial.

Figure 5 shows the estimated walking paths for all viable trials of each of the four floor obstacle configurations. All data was considered for this analysis as long as the cups on the table and at least one of the cups on the floor was visible within the scene camera's images. This was usually so until the second (yellow) obstacle on the floor disappeared below the scene camera's view, but four (of the 800) trials are not shown because there were large gaps in the data due to the participant rotating his or her head too far to one side (so not enough cups were visible in the scene camera's images). The figure shows that there was quite a lot of variability in participants’ paths. Not surprisingly, they usually passed to the right of the cups on the floor when the cups were both on the left side (Figure 5A), and to the left of cups when they were both on the right side (Figure 5D). Participants often passed between the obstacles when they were on opposite sides (Figures 5B and 5C). But sometimes participants took a longer path, curving around the obstacles, and sometimes they stepped over obstacles, so it looks as if they walked straight through them.

There are occasional instances where the participant's position is implausible. This suggests that our automatic detection algorithm mistook some other item in the room for a cup (and in one case it did because we had placed the red cup at the wrong position; this was not accounted for in the analysis because we do not know the cup's true position). We did not exclude trials with implausible walking paths from our analysis, because although there are quite a few cases in which the paths were implausible at the very beginning or end, the paths are probably quite reliable when entering the room and between the door and the turquoise cup, which is the main part in relation to our analysis of where on the floor the participants were looking.

**Figure 6.**
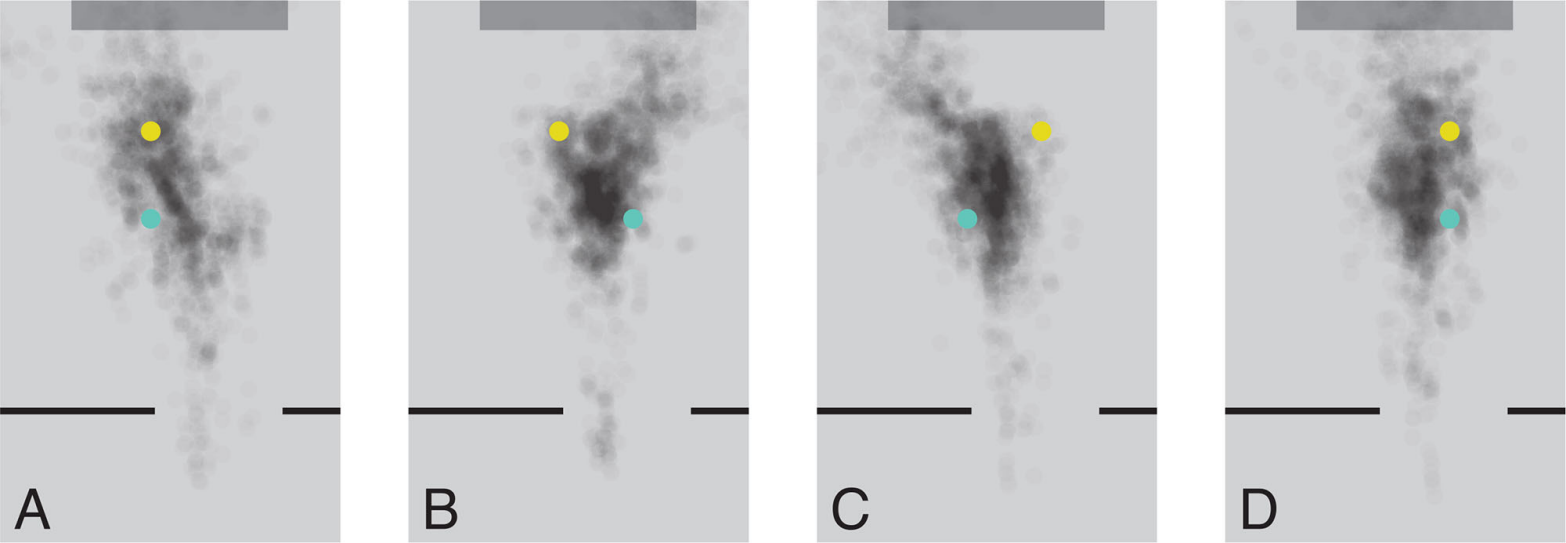
Estimated gaze positions on the floor for each of the four possible floor obstacle configurations. Plots show a top view of the room, from the door (bottom) to the table (top). The *yellow* and turquoise disks are the two floor obstacles, so each of the panels **A**–**D** represents a single floor obstacle configuration. Each line represents a single trial.

Figure 6 shows the estimated gaze positions on the floor for each floor obstacle configuration. Only moments at which gaze was directed at the floor or the cups on the floor were considered. Gaze was mainly directed at the floor near where the walking paths crossed the cups (compare Figures 5 and 6). As we had already seen in Figure 4, participants did not mainly look at the floor obstacles themselves. When both floor obstacles were on the left, participants’ gaze was less consistently directed towards a certain region of the floor. This might be because in this case it was relatively easy to walk to the table without knocking over the floor obstacles, because in this case the door and the target cup were both to the right of both floor obstacles, so participants could just walk straight into the room. Overall, Figures 5 and 6 confirm what Figure 4 had suggested: that when participants’ gaze was directed toward the floor, it was mainly toward critical positions for foot placement along their walking path, rather than toward the obstacles themselves.

**Figure 7.**
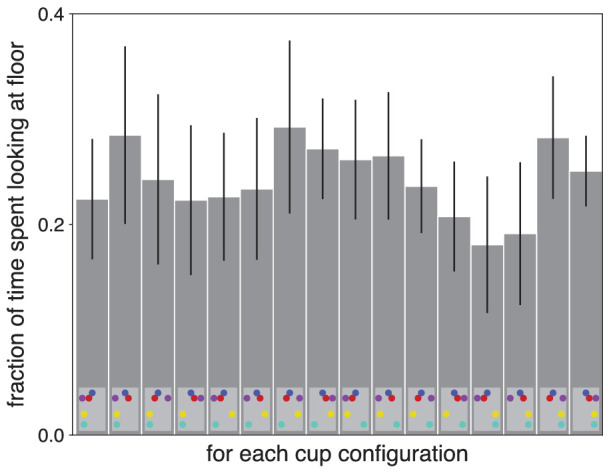
Total time spent looking at the floor for each of the 16 possible configurations of the cups. The configurations are indicated by the schematic top views of the scene at the bottom of each bar (*light gray* areas). Values are averages with 95% confidence intervals across participants.

If gaze is directed at the floor to guide foot placement at critical positions, one might expect people to look at the floor longer when the two floor obstacles are at opposite sides, because when they are at opposite sides participants usually walked in between them (see lines in the central panels of Figure 5). Moving one's feet between the obstacles without knocking them over probably requires more precise guidance than just ensuring to move far enough to the side. However, participants did not systematically spend more time looking at the floor when the two floor obstacles were at opposite sides than when they were at the same side (Figure 7). The only systematic effect that we found was that participants looked at the floor slightly longer when the red and yellow cups were at opposite sides (24.8% of the time) than when they were at the same side (24.2% of the time; interaction between the positions of the red and yellow cups in the analysis of variance: *F*(1, 9) = 31.22, *p* < 0.001). Maybe having the red cup at one side made it advantageous to approach the table slightly from the other side, which is closer to the last obstacle on the floor if the cups are at opposite sides, increasing the need to look at the floor.

## Discussion

We were interested in how participants allocate their gaze when walking to grasp and manipulate an object if they have to avoid obstacles along their walking path as well as near that object (the cup of water). Having an obstacle next to the cup of water made it more difficult to reach and grasp that cup, which might prompt participants to not only look at that cup but to also look at the nearby obstacle to consider the best way to approach the cup of water. We were particularly interested in whether participants would mainly look at the obstacles along their walking path as they enter the room, and only later look at the cups on the table, as one might expect from some previous findings (Johansson et al., 2001; Patla & Vikers, 1997). Or whether they would immediately mainly look at the object that is most important for the task or that requires the most precision, here the cup of water that they have to pick up, as one might expect from other previous findings (Rothkopf, Ballard, & Hayhoe, 2007; Voudouris et al., 2016).

Gaze allocation varied to some extent both within and between participants, with one of the most conspicuous differences between participants being the frequency with which they blinked (dark green in Figure 3), but there were also some evident common trends. Well before entering the room, most participants already often looked at the cups on the table (Figure 4). At that distance from the table, we often cannot differentiate between looking at the target cup or the surrounding obstacle. Knowing these cups’ positions might be important when entering the room, because determining the best place to stand for grasping the cup of water and even for later pouring its contents into the purple cup can be useful for determining how to approach the table. People are known to aim for certain hand and arm configurations at the moment of grasping (Voudouris, Brenner, Schot, & Smeets, 2010; but see also Klein, Maiello, Fleming, & Voudouris, 2021) and to consider obstacles in the paths of the fingers and of the arm when doing so (Voudouris, Smeets, & Brenner, 2012). That is probably why people look at objects that they intend to grasp (Land et al., 1999; Voudouris et al., 2018) and at potential obstacles close to them (Grant, 2015) before initiating movements toward them. Here they do so well in advance.

As participants entered the room, they were often looking at the floor (Figure 4). They mostly looked at positions near the floor obstacles (Figure 6). They were most likely to look at such positions when they were about 2 m (about two steps) from them (Figure 4), which is consistent with earlier findings about when one looks where one will place one's foot when walking on challenging terrains (Matthis et al., 2018). They presumably looked where they intended to place their feet at the most critical moment for foot placement, so when close to the cups on the floor (Figure 5), despite placing the foot not being particularly challenging in our study, there being nothing particularly informative to see at those positions, and those positions not being particularly salient. It was already known that people look where they place their feet when this has to be done precisely, either because the task is to place them at certain places (Hollands & Marple-Horvat, 2001) or because the terrain requires precise foot placement (Matthis et al., 2018). But foot placement was not very challenging in our study, so it is interesting to see that our participants mainly looked at the floor rather than at the obstacles when the only real constraint on foot placement was not to knock over the obstacles. The configuration of the obstacles on the floor (and of the obstacle and destination cup on the table) changed across trials, so participants could not simply repeat the same sequence of walking and grasping movements. They must therefore have relied on peripheral visual input and possibly on their memory of the obstacles’ positions from brief glimpses earlier on (for instance at the beginning of the trial) to avoid knocking the floor obstacles over as they crossed them.

In studies in which gaze was found to specifically be directed at obstacles, the obstacles were obstructing the complete path so that participants had to step over them (Patla & Vikers, 1997). In our study, participants could choose to cross to the side of, or between, the obstacles on the floor, as well as to step over them. We therefore interpret our findings as support for the idea that gaze is primarily directed at critical points along the intended path. If participants step over an obstacle, the position of the obstacle is a critical position along the path, so gaze is directed at the obstacle. If participants walk around an obstacle, the obstacle is not on the path. We here show that in that case gaze is directed at the path near the obstacle. However, we know that this is not always the case. When people are specifically instructed to avoid virtual obstacles that they can walk around but not step over, they do spend quite some time looking directly at the obstacles (Rothkopf et al., 2007), especially if their positions are uncertain (Tong et al., 2017). One difference is that, in those studies, avoiding the obstacles was part of the navigation task, whereas in our study participants had to pass the obstacles on the floor on their way to the table, but the main task was to pour the water from the blue cup into the purple one. This might make a difference, because gaze when navigating obstacles and targets was influenced by the imposed task (Rothkopf et al., 2007). It is not yet clear when exactly people look at obstacles rather than relying on peripheral vision to avoid them.

An obvious concern in any eye movement study, and one that is particularly relevant in studies performed under unconventional circumstances (while walking), is whether the measurements are accurate and precise enough to justify the analysis and conclusions. A very attractive feature of the Pupil Invisible eye tracker is that it provides quite precise measurements (Ghiani et al., 2024), even when walking (on stairs; Ghiani et al., 2023): a standard deviation of about 1°. In our study, estimates of gaze direction need to be combined with estimated positions of the relevant cups based on image analysis. We used the data from when participants were pouring water into the destination cup to estimate the precision of the gaze estimates that are relevant for our study. We found a standard deviation of about 1.8° in the horizontal direction. This is the median value across separate standard deviations determined on many trials. We used the median because participants could sometimes briefly look away from the pouring water, in which case the standard deviation became very high. Such high standard deviations represent actual deviations of gaze, rather than measurement errors, so they should not be included in our estimate of the precision of our gaze analysis. We found a larger standard deviation in the vertical direction (but as explained earlier, in this case the variability probably includes actual changes in gaze that occur when participants do not look away, as well as errors in the eye tracker and in judging the position of the center of the cup). Thus the precision of the eye tracker is not evidently the limiting factor in our analysis. The image analysis presumably contributes substantially as well. This is not too surprising, because we identified the cups on the basis of color, so, for instance, specular reflections or occluded parts are not considered to be part of the cup. We considered participants to be looking at one of the cups whenever we were not quite certain that they were not (the threshold distance of 4°).

Although this way of analyzing gaze assumes that participants are looking at the cups whenever in doubt, it might still sometimes miss instances at which gaze is directed at the cups when the cups are nearby. When participants are standing near the cups on the table, the cups images (on the screen and on the retina) are quite large (Figure 2B). In that case we might sometimes misclassify looking at the edge of the cup (for instance looking where one intends to place one's index finger; Voudouris et al., 2016) as looking at the table, because the edge of the cup might be up to several degrees from what we judge to be the center of the cup. In addition, the Pupil Invisible eye tracker has the scene camera placed at the side of the glasses, which gives rise to a systematic error when looking at objects that are very nearby. Thus when standing nearby the cups we cannot be certain that we are not underestimating how often gaze is directed at the cups. When the cups are further away, this is not a problem because the images of the cups are much smaller. Importantly, such errors cannot account for any of the findings on which we base our conclusions.

For Figures 5 and 6, we used the configuration of the cups in the image to estimate the participant's position in the room, and used the judged positions of the participant and the cups, together with the participant's eye height, to evaluate where exactly on the floor the participant was looking. Although we could not judge the participant's position very precisely, the error in determining where they were looking on the floor is not too imprecise, because the ordinal position relative to the cups is maintained. The only factor that is influenced by considering the participant's viewpoint is the relative position with respect to the cups, which depends to some extent on where the participant is situated because it depends on the viewing angle with respect to the ground surface. Thus variability in estimating the participant's position may have introduced some additional variability in the gaze positions on the ground (Figure 6), but less variability would not change our conclusions. Although our measures are certainly not perfect, we believe they are good enough to support our conclusions. Note that the analyses of where participants were looking in Figures 3 and 4 do not depend on judging the participant's position. Only the precise horizontal positions in the figures (the distance from the blue cup) do.

During the experiment, gaze shifted systematically between looking at different cups as they became more relevant for the upcoming actions: walking, grasping, pouring. The only exception is at the very beginning of the trials, when participants often looked at items on the table although they would only really become relevant much later. This is consistent with earlier findings. When asked to prepare breakfast in an unfamiliar setting, people first look around to explore the available items and their positions (Hayhoe, Shrivastava, Mruczek, & Pelz, 2003). Likewise, when entering a room for the first time, people first look around for relevant objects. They look around less, presumably using knowledge obtained the first time, when entering the same room again (Li, Aivar, Kit, Tong, & Hayhoe, 2016). In our experiment, the positions of the obstacles and of the cup into which the water had to be poured changed between trials. Therefore participants probably looked around to localize the cups and determine a suitable walking path before walking into the room. They then used their memory of the cups’ positions and of the path they had determined based on glimpses at the onset of the trial, together with peripheral visual information, to guide them to the table without directing their gaze to all the cups again.

When performing goal-directed arm movements, people usually look at the target object (Cámara et al., 2018; Danion & Flanagan, 2018; Grant, 2015; Voudouris et al., 2016), although they do look at obstacles when maneuvering a hand-held object around them (Johansson et al., 2001). When walking, people look at the ground more often when visual guidance of foot placement is more important (Hollands & Marple-Horvat, 2001; Matthis et al., 2018). We here show that when walking into a room to grasp an object, people do not necessarily look at obstacles on their path to the object, but primarily look at the places at which interacting with the environment requires some precision, even if there is nothing in particular to see at those positions. Thus, people sequentially direct their gaze to where precision is required, relying on peripheral vision and memorized information to cope with challenges at other positions that are relevant for the task at hand.

## References

[bib1] Brenner, E., & Smeets, J. B. J. (2007). Flexibility in intercepting moving objects. *Journal of Vision**,* 7(5), 14. 18217854 10.1167/7.5.14

[bib2] Brenner, E., & Smeets, J. B. J. (2011). Continuous visual control of interception. *Human Movement Science**,* 30(3), 475–494. 21353717 10.1016/j.humov.2010.12.007

[bib3] Brouwer, A.-M., Franz, V. H., & Gegenfurtner, K. R. (2009). Differences in fixations between grasping and viewing objects. *Journal of Vision*, 9(1), 18. 19271888 10.1167/9.1.18

[bib4] Cámara, C., de la Malla, C., López-Moliner, J., & Brenner, E. (2018). Eye movements in interception with delayed visual feedback. *Experimental Brain Research**,* 236(7), 1837–1847. 29675715 10.1007/s00221-018-5257-8 PMC6010481

[bib5] Danion, F. R., & Flanagan, J. R. (2018). Different gaze strategies during eye versus hand tracking of a moving target. *Scientific Reports*, 8(1): 10059. 29968806 10.1038/s41598-018-28434-6 PMC6030130

[bib6] Dominguez-Zamora, F. J., Gunn, S. M., & Marigold, D. S. (2010). Adaptive gaze strategies to reduce environmental uncertainty during a sequential visuomotor behavior. *Scientific Reports**,* 8(1), 14112. 10.1038/s41598-018-32504-0 PMC6148321 30237587

[bib7] Franchak, J. M., & Adolph, K. E. (2010). Visually guided navigation: head-mounted eye-tracking of natural locomotion in children and adults. *Vision Research*, 50(24), 2766–2774. 20932993 10.1016/j.visres.2010.09.024 PMC3013502

[bib8] Ghiani, A., Van Hout, L. R., Driessen, J. G., & Brenner, E. (2023). Where do people look when walking up and down familiar staircases? *Journal of Vision**,* 23(1): 7. 10.1167/jov.23.1.7 PMC9840440 36633872

[bib9] Ghiani, A., Amelink, D., Brenner, E., Hooge, I. T. C., & Hessels, R. S. (2024). When knowing the activity is not enough to predict gaze. *Journal of Vision**,* 24(7), 6. 10.1167/jov.24.7.6 PMC11238878 38984899

[bib10] Grant, S. (2015). Gaze-grasp coordination in obstacle avoidance: Differences between binocular and monocular viewing. *Experimental Brain Research*, 233(12), 3489–3505. 26298046 10.1007/s00221-015-4421-7

[bib11] Hayhoe, M. M., Shrivastava, A., Mruczek, R., & Pelz, J. B. (2003). Visual memory and motor planning in a natural task. *Journal of Vision**,* 3(1), 49–63. 12678625 10.1167/3.1.6

[bib12] Hollands, M. A., & Marple-Horvat, D. E. (2001). Coordination of eye and leg movements during visually guided stepping. *Journal of Motor Behavior**,* 33(2), 205–216. 11404215 10.1080/00222890109603151

[bib13] Ioannidou, F., Hermens, F., & Hodgson, T. L. (2017). Mind your step: the effects of mobile phone use on gaze behavior in stair climbing. *Journal of Technology in Behavioral Science**,* 2(3), 109–120. 29387779 10.1007/s41347-017-0022-6 PMC5770487

[bib14] Johansson, R. S., Westling, G., Backstrom, A., & Flanagan, J. (2001). Eye–hand coordination in object manipulation. *Journal of Neuroscience*, 21(17), 6917–6932. 11517279 10.1523/JNEUROSCI.21-17-06917.2001 PMC6763066

[bib15] Klein, L. K., Maiello, G., Fleming, R. W., & Voudouris, D. (2021). Friction is preferred over grasp configuration in precision grip grasping. *Journal of Neurophysiology**,* 125(4), 1330–1338. 33596725 10.1152/jn.00021.2021

[bib16] Land, M., Mennie, N., & Rusted, J. (1999). The roles of vision and eye movements in the control of activities of daily living. *Perception*, 28(11), 1311–1328. 10755142 10.1068/p2935

[bib17] Li, C. L., Aivar, M. P., Kit, D. M., Tong, M. H., & Hayhoe, M. M. (2016). Memory and visual search in naturalistic 2D and 3D environments. *Journal of Vision**,* 16(8), 9. 10.1167/16.8.9 PMC4913723 27299769

[bib18] Mann, D. L., Spratford, W., & Abernethy, B. (2013). The head tracks and gaze predicts: How the world's best batters hit a ball. *PLoS ONE*, 8(3), e58289. 23516460 10.1371/journal.pone.0058289 PMC3596397

[bib19] Marotta, J. J., & Graham, T. J. (2016). Cluttered environments: differential effects of obstacle position on grasp and gaze locations. *Canadian Journal of Experimental Psychology**.* 70(3), 242–247. 26751082 10.1037/cep0000079

[bib20] Matthis, J. S., Yates, J. L., & Hayhoe, M. M. (2018). Gaze and the control of foot placement when walking in natural terrain. *Current Biology**,* 28(8), 1224–1233. 29657116 10.1016/j.cub.2018.03.008 PMC5937949

[bib21] Neggers, S. F., & Bekkering, H. (2000). Ocular gaze is anchored to the target of an ongoing pointing movement. *Journal of Neurophysiology*, 83(2), 639–651. 10669480 10.1152/jn.2000.83.2.639

[bib22] Patla, A. E., & Vikers, J. N. (1997). Where and when do we look as we approach and step over an obstacle in the travel path? *Neuroreport**,* 8(17), 3661–3665. 9427347 10.1097/00001756-199712010-00002

[bib23] Riek, S., Tresilian, J. R., Mon-Williams, M., Coppard, V. L., & Carson, R. G. (2003). Bimanual aiming and overt attention: One law for two hands. *Experimental Brain Research*, 153(1), 59–75. 12923603 10.1007/s00221-003-1581-7

[bib24] Rothkopf, C. A., Ballard, D. H., & Hayhoe, M. M. (2007). Task and context determine where you look. *Journal of Vision*, 7(14), 16. 18217811 10.1167/7.14.16

[bib25] ‘t Hart, B. M., Schmidt, H., Klein-Harmeyer, I., & Einhaeuser, W. (2013). Attention in natural scenes: contrast affects rapid visual processing and fixations alike. *Philosophical Transactions of the Royal Society of London. Series B, Biological Sciences**,* 368(1628), 20130067. 24018728 10.1098/rstb.2013.0067 PMC3758209

[bib26] Tong, M. H., Zohar, O., & Hayhoe, M. M. (2017). Control of gaze while walking: task structure, reward, and uncertainty. *Journal of Vision**,* 17(1), 28. 28114501 10.1167/17.1.28 PMC5256682

[bib27] Tuhkanen, S., Pekkanen, J., Wilkie, R. M., & Lappi, O. (2021). Visual anticipation of the future path: predictive gaze and steering. *Journal of Vision**,* 21(8), 25. 10.1167/jov.21.8.25 PMC8399320 34436510

[bib28] Voudouris, D., Smeets, J. B. J., Fiehler, K., & Brenner, E. (2018). Gaze when reaching to grasp a glass. *Journal of Vision**,* 18(8), 16. 10.1167/18.8.16 30167674

[bib29] Voudouris, D., Smeets, J. B. J., & Brenner, E. (2016). Fixation biases towards the index finger in almost-natural grasping. *PLoS ONE*, 11(1), e0146864. 26766551 10.1371/journal.pone.0146864 PMC4713150

[bib30] Voudouris, D., Smeets, J. B. J., & Brenner, E. (2012). Do obstacles affect the selection of grasping points? *Human Movement Science**,* 31(5), 1090–1102. 22698834 10.1016/j.humov.2012.01.005

[bib31] Voudouris, D., Brenner, E., Schot, W. D., & Smeets, J. B. J. (2010). Does planning a different trajectory influence the choice of grasping points? *Experimental Brain Research**,* 206(1), 15–24. 20820763 10.1007/s00221-010-2382-4 PMC2938418

